# Evaluation of high performance liquid chromatography (HPLC) pattern and prevalence of beta-thalassaemia trait among sickle cell disease patients in Lagos, Nigeria

**DOI:** 10.11604/pamj.2014.18.71.4239

**Published:** 2014-05-22

**Authors:** Titilope Adeyemo, Oyesola Ojewunmi, Ajoke Oyetunji

**Affiliations:** 1 Department of Haematology and Blood transfusion, College of Medicine, University of Lagos, P.M.B 12003, Surulere, Lagos, Nigeria; 2 National Sickle Cell Centre, Surulere, Lagos, Nigeria

**Keywords:** Sickle cell disease, thalassaemia, microcytosis, HPLC, haemoglobin variants, foetal haemoglobin, Nigerian patients, hypochromia

## Abstract

**Introduction:**

Sickle cell disease (SCD) is the most common inherited disorder of haemoglobin worldwide. This study evaluated the chromatographic patterns and red blood cell indices of sickle cell patients to determine the co-inheritance of other haemoglobin(Hb) variants and β-thalassaemia trait.

**Methods:**

Red cell indices, blood film, sickle solubility test, Hb electrophoresis using alkaline cellulose acetate membrane, and chromatographic patterns using Bio Rad HPLC Variant II were evaluated for 180 subjects.

**Results:**

Based on low MCV <76fL and MCH<25 pg, in the presence of elevated A_2_ >4.0% on HPLC and Hb variants eluting outside the S and C windows, at least four haemoglobin phenotypes (SS: 87.7%; SC: 1.1%; SD Punjab: 0.6%; Sβ-thalassemia: 10.6%) were identified. Mean Hb F% was 8.1±5.1 (median 7.65) for Hb SS and 6.03±5.2 (median 3.9) for Hb Sβ-thalassemia trait. Majority of Hb SS (69.1%) had Hb F% less than 10 while 27.6% had 10-19.9 and 3.2% had ≥ 20. Mean Hb F% was higher in female Hb SS (9.55±5.09; mean age 7.4±3.8 years) than the males (7.63±4.80; mean age 6.9±3.8 years) (P=0.02). A borderline significant negative correlation between age and Hb F levels among Hb SS subjects (r= -0.169 P=0.038) was also observed.

**Conclusion:**

Our data suggests that α and β- thalassaemia traits, and other haemoglobin variants co-exist frequently with SCD in our population

## Introduction

Haemoglobinopathies are inherited disorders characterised by either an abnormality in the structure of haemoglobin such as in sickle cell anaemia or reduced production of one or more globin chains in thalassaemia [1]. The latter is generally classified into two: alpha thalassaemia - usually caused by deletions of one or both of the duplicated α- genes and beta thalassaemia which is typically caused by a point mutation in one of the β-globin genes resulting in a reduced or absent β-globin chain synthesis [2]. Sickle cell disease (SCD) is the most common, accounting for about 70% of the world's major haemoglobinopathies [3]. It comprises sickle cell anaemia and other compound heterozygous state such as haemoglobin SC disease, Sβ-thalassaemia, and SD-Punjab. About 5% of the world's populations are carriers of genes responsible for haemoglobinopathies and about 300,000 children are born annually with haemoglobin disorders. Approximately 70% of these cases occur in sub- Sahara Africa [4]. Due to the high prevalence of sickle cell trait at 23.7%, the frequency of sickle cell anaemia in Nigeria is about 20 per 1000 births resulting in about 150,000 babies being born each year with the disorder [4]. This makes Nigeria the country with the largest burden of sickle cell disease globally with 4.1% of the population being affected [5]. Current methods used by laboratories in the evaluation of haemoglobin disorders include sickle solubility test, alkaline and acid electrophoresis, isoelectric focusing (IEF), high performance liquid chromatography (HPLC), capillary electrophoresis, globin chain electrophoresis and DNA analysis/protein analysis [2]. However, it is important to stress that in conjunction with any of these methods, evaluation of the peripheral blood smear, as well as correlation with the results of a full blood count (FBC) are very important as many of the clinically significant haemoglobin disorders show characteristic peripheral blood findings, and are often co-inherited. This present study evaluated the high performance liquid chromatographic patterns and red cell indices of sickle cell disease patients to determine the co-inheritance of other haemoglobin (Hb) variants and β-thalassaemia traits in SCD patients in Nigeria.

## Methods

### Patients and clinical procedures

This was a prospective study of 180 Sickle cell disease (SCD) patients in steady state who presented at the haemoglobin reference laboratory of National Sickle Cell Centre, Lagos, Nigeria. Patients on hydroxyurea and those who have had blood transfusion at most four months prior to recruitment for the study were excluded. Ethical approval was granted by the Health Research Ethics Committee of the Lagos University Teaching Hospital, Lagos, Nigeria. 5 ml venous blood sample was collected into EDTA anticoagulant bottles after obtaining written informed consent from parents/guardians.

### Laboratory Procedures

Red cell indices were evaluated using an automated haematology analyser (Mindray, BC-2800). Microcytic, hypochromic anaemia is pivotal to diagnosis of β-thalassaemia trait with MCV and Hb A_2_ being significant diagnostic elements [6]. In this study, subjects with MCV<76fL and MCH<25pg, in the presence of elevated A_2_ >4.0% on HPLC were presumed to have Sβ-thalassemia trait while those with A_2_ <3.5% and borderline A_2_ (<3.5-4.0%) with MCV<76fL and MCH<25pg are presumed to have either an iron deficiency or a co-existing α-thalassaemia [7].

Solubility test was performed by standard method as described by Dacie and Lewis and conducted with both positive and negative controls. The presence of a red precipitate indicated Hb S [8]. Cellulose acetate electrophoresis was done on samples and controls also by standard methods as described by Dacie and Lewis. Blood smear was stained with Leishman's stain and examined microscopically for morphology of the red cells [8]. High Performance Liquid Chromatograph of samples was carried out using Bio-Rad Laboratories, Hercules, CA. The chromatographic patterns were evaluated for the identification and quantification of different Hb variants. Each haemoglobin variant has its characteristic retention time. Retention time is the elapsed time from the sample injection to the apex of a haemoglobin peak. The “windows” are established ranges in which common variants have been observed to elute using the Variant beta - thalassaemia short program. The printed chromatogram shows all the haemoglobin variants eluted, the retention times, the areas of the peaks and the values (%) of different haemoglobin components. If a peak eluted at a retention time that is not pre-defined, it is labelled as an unknown.

### Statistical analysis

Data was analyzed using SPSS version 21 (IBM Inc.) and presented as mean ± standard deviation. Independent student's t-test was used to compare means of two variables while one-way ANOVA was used to compare mean difference among three variables. Pearson correlation was used to test the relationship between variables. P-value *<*0.05 was considered statistically significant.

## Results

The mean age of the 180 study participant was 7.08 ± 3.81 years, median 6.0 years and age range 2-15 years. There were 70 females (38.9%) to 110 males (61.1%). The Solubility test was positive for all samples confirming the presence of haemoglobin S. Haemoglobin electrophoresis showed 178 (98.9%) of the patients are homozygous for Hb S and 2 (1.1%) were heterozygous for SC. However, using the high performance liquid chromatographic patterns and HbA2 levels >4.0% and red cell indices (MCV<76Fl, MCH<25pg), four haemoglobin phenotypes; 158 Hb SS (87.7%); 2 Hb SC (1.1%); 1 SD-Punjab (0.6%) and 19 Sβ-thalassemia trait (10.6%) were identified in this study. Table 1 shows HPLC patterns and red cell indices of the study participants. The mean MCV and MCH in Sβ-thalassemia trait (69.7±5.3, 21.6±1.7) were significantly lower (*P<0.05*) than Hb SS (79.2±8.1, 28.4±2.6). Mean Hb A_2_ of Sβ-thalassemia trait (5.14±1.3) was also significantly higher (*P<0.05*) than Hb SS (3.76±2.53). Mean Hb F% was 8.1±5.1 (median 7.65; range 1.2-25.5%) for Hb SS and 6.03±5.2 (median 3.9, range 1-21.4%) for Hb Sβ-thalassemia trait. Mean HbF% was higher in female Hb SS (9.55±5.09; mean age 7.4±3.8 years) than the males (7.63±4.80; mean age 6.9±3.8 years) (*P<0.05*). So is Hb A_2_ significantly higher in females than in male Hb SS. Patients with Sβ-thalassemia trait did not show any significant differences in HbF% and HbA_2_% between the sexes (Table 2). Higher values of Hb F% were observed in children aged less than 5 years (9.23±5.3), but no statistical difference was observed between the different age groups (Table 3). Hb F% in Sβ-thalassemia trait reduces significantly with age (Table 3).

**Table 1:** Haemoglobin chromatographic pattern and red cell indices

	SS (158)	Sβ-Thal traits (19)	SC (2)	SD-Punjab (1)
**Hb F (%)**	8.05±5.07	6.03±5.20	0.45±0.07	-
**Hb A**_**2**_ **(%)**	3.76±2.53	5.14±1.27^a^	4.30±2.40	3.3
**Hb S (%)**	82.90±7.69	84.13±6.94	49.80±1.98^ab^	39.5
**Hb (g/dL)**	7.35±0.95	6.76±1.25	8.80±0.85^ab^	10.5
**PCV (%)**	22.17±3.13	21.72±4.04	28.50±3.54^ab^	25
**MCV (fL)**	79.17±8.05	69.68±5.32^c^	67.3±6.08	80.9
**MCH (pg)**	28.42±2.61	21.59±1.71^c^	22.45±1.48^a^	23.10
**MCHC (g/dL)**	32.57±1.37	28.18±1.03^c^	29.00±0.42^a^	28.60

a *P*<0.05 compared to Hb SS group.

b *P*<0.05 when compared to Sβ thal traits.

c *P*<0.001 compared to Hb SS group.

**Table 2:** HbF% and HbA_2_ according to sex

	Sex	Hb SS	p-value	Hb Sβ-Thal	p-value
**Hb F**	M	7.63±4.80	0.02	5.68±5.80	0.63
	F	9.55±5.09		6.78±3.87	
**Hb A**_**2**_	M	3.14±1.89	0.02	5.18±1.36	0.81
	F	4.13±3.37		5.05±1.13	

**Table 3:** HbF% and A_2_ according to age

		≤5	6-10	11-15
**Hb F**	HbSS	9.23±5.3	7.71±4.6	7.82±5.0
	Sβ-thal	9.99±6.9	3.94±1.9*	3.63±1.8*
**Hb A**_**2**_	HbSS	3.29±2.7	3.78±2.6	3.60±2.6
	Sβ-thal	5.80±1.4	4.93±1.2	4.56±0.9

* P<0.05.

We observed a borderline significant negative correlation between age and Hb F levels among Hb SS subjects (r= -0.169 P=0.038). HbA_2_ did not show any significant difference with age in Hb SS and Sβ-thalassemia trait. Majority of Hb SS (69.1%) had HbF% less than 10 while 27.6% had 10-19.9 and 3.2% had ≥ 20. Association between foetal haemoglobin distribution and age among Hb SS as shown in Table 4 did not show any significance (P=0.889). Thirty-five of 158 Hb SS (22.1%) had MCV_2_% (mean 2.81±1.38) while the remaining 6 had borderline HbA_2_ (3.5-4.0%) which is suggestive of dβ thalassaemia.

**Table 4:** Foetal haemoglobin distribution according to age among Hb SS

Age	Hb F				P-value
	0-2	2-9.9	10-19.9	≥20	X^2^=2.265 df=6 p-value=0.894
≤5	4 (2.6%)	37 (24.3%)	21 (13.8%)	3 (2.0%)	
6-10	4 (2.6%)	38 (25%)	14 (9.2%)	1 (0.7%)	
11-15	2 (1.3%)	20 (13.2%)	7 (46%)	1 (0.7)	

## Discussion

Haematological characteristics and clinical severity of SCD are heterogeneous and are associated with environmental and genetic factors that include variation in HbF level, the haplotype locale that is linked to the β-globin gene and the co-inheritance of - α-thalassaemia and other Hb variants [9]. Full blood count and the red cell indices are essential in the preliminary investigation of haemoglobinopathies. Though cellulose electrophoresis at alkaline pH is highly reproducible and is able to separate haemoglobin variants within a very short time, it cannot be used to separate Hb S, Hb D, and Hb G at this pH because they co-migrate. Hb A2, Hb C, Hb E, and Hb O-Arab also co-migrate at alkaline pH. Sickle solubility test detects the presence of haemoglobin S by precipitation of the insoluble haemoglobin variant creating a cloudy, turbid suspension in a prepared test solution. One sample showed a positive solubility test like the AS control but on cellulose acetate electrophoresis at alkaline pH, showed only one band in the S region. However, the chromatographic pattern for co-inheritance of Hb S and D was clearly shown. This Hb variant and its co-inheritance with Hb S are rare but present in Nigerian population. Hb D is seen in blacks and people from India and sporadically in other races. Therefore, accurate diagnosis of haemoglobinopathies which has implications for genetic counselling cannot be overemphasized in Nigeria.

The Mean MCV reported in this present study among Hb SS patients is in agreement with Omoti and Akinbami et al., who reported 79.2 fL and 81.52 fL respectively [10, 11]. Mean MCV, MCH, and MCHC were significantly reduced (*P*<0.05) in patients with β-thalassaemia trait compared to the Hb SS patients (Table 1). The ranges of MCV, MCH, and Hb A2 observed in this study are in agreement with what Old described for carriers of β-thalassaemia [12]. Several studies have shown that the complement of MCH11-13]. Quantitative evaluation of Hb A_2_ by elution, spectrophotometry or densitometric scanning after cellulose acetate electrophoresis or micro-column chromatography is not always precise [14]. HPLC has replaced alkaline electrophoresis as the primary screening method of haemoglobinopathies. The reliability and reproducibility of results has made HPLC the method of choice for quantifying Hb F and Hb A_2_ for laboratory diagnosis of haemoglobinopathies [15]. Elevated HbA_2_ is the most significant parameter in the identification of β-thalassaemia carriers. However, in some cases, the level of HbA_2_ is not typically elevated and some difficulties may arise in making the diagnosis. For these reasons, the quantification of HbA_2_ has to be performed with great accuracy and the result interpreted together with other haematological and biochemical parameters [7]. This is also difficult in the presence of Hb S when HbA_2_ measured by HPLC is often elevated because of co-elution with glycated Hb S [16]. However, when Hb S variant is present, and Hb A is reduced or absent, percentage Hb A_2_, Hb F, and FBC parameters may be useful for distinguishing between homozygosis for Hb S and combination of Hb S and β- thalassaemia (hemizygosis) [17].

Phenotypic heterogeneity of sickle cell disease is strongly modulated by foetal haemoglobin [18]. Reduced rate of acute painful episodes, leg ulcers, osteonecrosis, acute chest syndromes, and reduced disease severity has all been associated with elevated levels of Hb F while the association of complications like stroke and priapism with Hb F is unclear [19]. These could point to the fact that Hb F levels are influenced by β-globin haplotypes and genetic polymorphisms. For instance, Senegal and Arab-Indian haplotypes usually have the highest Hb F and mildest clinical presentations while Bantu haplotypes have the lowest Hb F and most severe clinical course; Benin haplotypes have intermediate levels of Hb F and moderate clinical course [20]. Varied foetal haemoglobin levels (2-9%) have been reported from different studies involving sickle cell patients in Nigeria [21–25]. The mean (±SD) HbF level of 8.05±5.07%, range 0.4 - 25.5% in our Hb SS population is comparable to that of previous studies in Nigeria which reported means ranging from of 7.4 - 9.5% [24, 25]. We suspect that discrepancies in the foetal haemoglobin levels estimated in Nigerian Sickle cell patients may be the consequence of method used in the various studies. For instance, alkali denaturation method of Hb F estimation may be subject to under estimation [14]. Studies from Congo, Uganda, and Saudi Arabia had reported 8.8%, 9.0% and 9.1% respectively [26, 27]. In our study, mean Hb F level was higher in Hb SS females than males with statistical significance (P=0.02). This is supported in other studies [21, 26, 28, 29]. It is not consistent with other studies that reported higher Hb F level in males than in females although with no statistical significance [22, 24]. It was surmised that hormonal effects of puberty might account for the difference observed in Hb F levels between females and males [28]. We observed a significant negative correlation between age and HbF levels among Hb SS subjects (r= -0.169, P=0.038). This is in agreement with the study of Olatunji [30]. We reported a higher proportion of subjects with elevated Hb F levels (32.3%) than a previous study in Nigeria (17%) but lower than what was reported from Uganda [25, 31]. Mean Hb A_2_ concentration observed among Hb SS patients in this study is higher than 2.4% reported by Fatunde and Scott-Emuakpor [25]. Falsely elevated levels of Hb A2 in the presence of Hb S when HPLC is employed may be responsible for this difference. Meanwhile, our mean Hb A_2_ is lower than 4.52% reported in another Nigeria study [10]. This higher value may suggest co-existing -βthalassaemia trait among the sickle cell population studied.

β-thalassaemia is predominantly found in the Mediterranean countries, the Middle East, Central Asia, India, North Coast of Africa and South America [32]. People with β-thalassaemia trait are usually clinically asymptomatic but sometimes have a mild anaemia [32]. About 10.6% of our study subjects had HbA_2_ >4.0% in the presence of microcytosis and hypochromia suggesting a co-existing β-thalassaemia. This is a much higher prevalence compared to that reported in previous studies [10, 33]. This could be because previous studies had used either only HbA2 level (_2_ estimation in this study (HPLC) also differs from that of previous study and may have overestimated the prevalence. To the best of our knowledge, this is the first time HPLC is reported in Nigeria as a primary screening of haemoglobinopathies. For this reason, a cutoff of HbA_2_ >4.0% was used in contrast to previous studies that used a cut off value of >3.5%. Besides, prevalence of β-thalassaemia trait in a Nigerian population with Hb AA which revealed 24% may lend credence to Sβ-thalassaemia trait reported in this study [34]. Although the presence of α-thalassaemia, commonly found in malaria endemic regions is of little clinical significance in Nigeria, predominance of α + thalassaemia (commonly -α^3.7^ deletion) in West African general population is quite known with prevalence of 45.5% from South-West Nigeria and 36-37% from two separate studies conducted in Ghana [35–37]. α-thalassaemia carriers are usually asymptomatic but can be slightly anaemic, microcytic and hypochromic. Co-inheritance of α-thalassaemia with Hb SS would also be present in the Nigerian population; this may show red cell indices with similar representation of β-thalassaemia trait [34] but when there is reduced/normal Hb A_2_ with reduced MCV and MCH with elevated Hb F (3-16%), δβ or γδβ thalassaemia may be present [14].

The distribution, clinical presentations, and haematological features of haemoglobinopathies in Nigeria have been previously investigated. In Nigeria, a resource limited country, evaluation of haemoglobinopathy is mostly limited to sickling/solubility test and alkaline electrophoresis. This unarguably underscores inaccurate diagnosis and limits our knowledge of the Hb variants present in our population. Therefore, it becomes imperative that evaluation of the peripheral blood smear, and red cell indices as well as confirmation of haemoglobin variant by alternative methods are very important in the diagnosis of sickle cell disease. This will provide accurate information for genetic risk assessment and counseling and adequate management of SCD.

## Conclusion

Our data suggest that α-thalassaemia, β- thalassaemia, and other Hb variants co-exist frequently with SCD in our population. Thus, presumptive diagnosis of haemoglobinopathy in Nigeria should transcend routine alkaline electrophoresis and solubility test and should include evaluation of full blood count, red cell indices, iron studies and HPLC as a method suited for the identification and quantification of other Hb variants while DNA analysis for definitive diagnosis will be good to have. The prevalence of Sβ- thalassaemia trait reported in this study is presumptive; a definitive study in a large cross-sectional study will be worthwhile.
